# The Influence of Diatomite on the Sound Absorption Ability of Composites

**DOI:** 10.3390/ma17184590

**Published:** 2024-09-19

**Authors:** Michał Łach, Eulalia Gliścińska, Agnieszka Przybek, Krzysztof Smoroń

**Affiliations:** 1 Materials Engineering, Faculty of Material Engineering and Physics, Cracow University of Technology, Jana Pawła II 37, 31-864 Kraków, Poland; 2 Textile Institute, Faculty of Material Technologies and Textile Design, Lodz University of Technology, Zeromskiego 116, 90-924 Lodz, Poland; klata@p.lodz.pl; 3 Specialized Mining Company GÓRTECH Sp. z o.o., 31-586 Kraków, Poland; k.smoron@diato.pl

**Keywords:** diatomite, sorbent of petroleum substances, calcination, sound absorption, composite

## Abstract

Diatomites are well-known mineral materials formed thousands of years ago from the skeletons of diatoms. They are found in many places around the world and have a wide range of applications. This article presents innovative research related to the possibility of using diatomite as a filler in composites to improve their sound absorption properties. The results of the study of the effect of diatomite processing (calcination) and its degree of fineness on the sound absorption coefficient of thermoplastic composites are presented. Three fractions of diatomite (0 ÷ 0.063 mm; 0.5 ÷ 3 mm; 2 ÷ 5 mm) and its variable mass proportion (0, 25, and 50 wt.%) were used. The composites were made with flax fibers as a reinforcement, polylactide as a matrix, and diatomite as an additional filler. This paper also presents the results of oxide chemical composition, diatomite mineral phase composition, morphology, and thermal conductivity coefficient of all diatomite fractions studied. In addition, the average particle size for diatomite powder was also determined. The most important of the studies was the determination of the acoustic properties of the aforementioned composites. As a result of the tests, it was found that the smallest fraction of diatomite particles and a variant without thermal treatment give the best effect in terms of sound absorption.

## 1. Introduction

**Figure 1:**
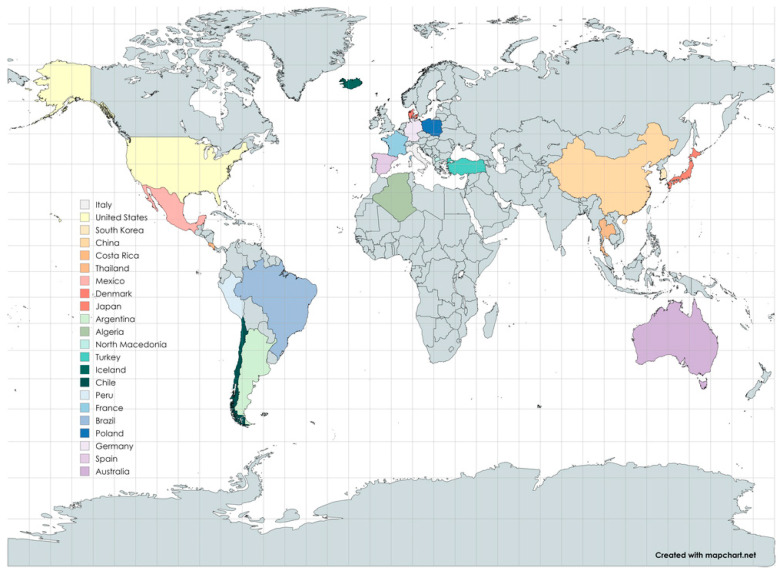
Map of countries where diatomite deposits are located and where diatomite mining is carried out (based on [3]).

Diatomite is a sedimentary rock composed of the fossilized skeletal remains of single-cell aquatic “algaes” known as “diatoms”. This unique form of silica has an elaborate honeycomb structure, peppered with thousands of tiny holes ranging from a few microns to submicron diameters. No other silica source, be it mined or artificially produced, presents such a structure. Some diatomite deposits are saltwater, but most are from freshwater sources. When ground, it results in an extremely low-density powder known as “diatomaceous earth” (DE), which has excellent absorption properties that are highly prized for filtration, agriculture, paints, plastics, cosmetics, and pharmaceuticals applications [1]. There are large, documented deposits of diatomites all over the world, with the largest amounts in the US, China, Spain, and Turkey [2]. The locations of diatomite deposits and where diatomite mining is carried out are shown in Figure 1 [3].

**Figure 2:**
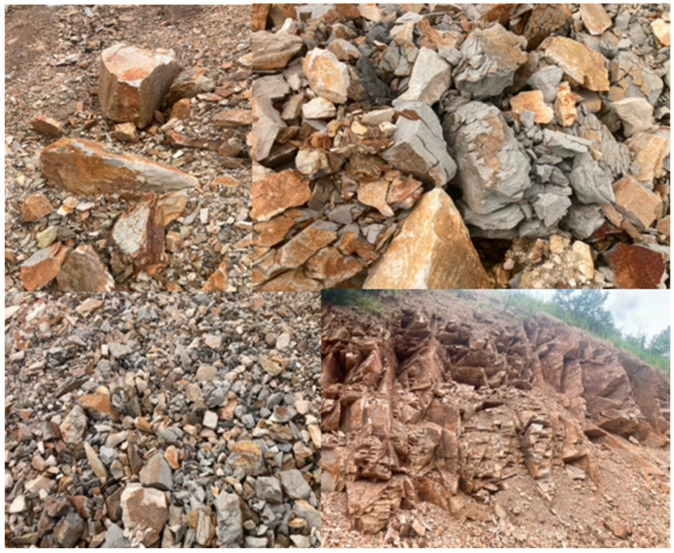
Diatomite mine in Poland. Appearance of diatomite rocks–different fractions and minerals visible.

As shown in Figure 1, diatomites also occur in Poland. In Poland, diatomite deposits are located in Podkarpacie, with the only active deposit located in Jawornik Ruski. Diatomites have a particularly high capacity to absorb petroleum substances and can reach a sorption capacity for these substances of up to 160 wt.%. [4]. Typical diatomites containing more than 80 wt.% SiO_2_ in their composition do not occur in Poland [1]. The composition of diatomites from mines in Poland is presented in articles [5,6]. The proven balance resources of diatomite rock are about 10 million tons. The diatomites mined in Poland, shown in Figure 2, are not homogeneous materials. There are different fractions in diatomite rock, and the way it is mined and processed causes the material to have slightly different contents of amorphous silica and other minerals. Nevertheless, it is finding increasing use in various industries [4].

Literature reports indicate that so far, the combination of diatomite with materials such as gypsum [7], zeolite [8], magnesium oxychloride cement [9,10], bitumen [11], rubber [12,13,14], polyethylene glycol [15,16], sodium acetate trihydrate (SAT)-acetamide (AC)-micron/nano aluminum nitride (AlN) composite [17], a sodium silicate solution [10], chitosan [18,19], PA11 [20], polyurethane [21], polyetherimide [22], polypropylene [23], poly (lactic acid) [24], poly (methyl methacrylate) [25,26], poly (acrylamide) [27], polyaniline [28], ethylene-vinyl acetate copolymer [29], and epoxy resin [30,31] has been tested.

Diatomite can be calcined [22], modified with bioactive substances [32], or modified with silane preparations in order to achieve better adhesion to the matrix material of the composite [14,15]. In order to improve the strength properties of the composite, in addition to the matrix and diatomite, e.g., polypropylene fibers [7] or multi-walled carbon nanotubes, MWCN [12] is used as the third component. Similarly, in the case of composite on the basis of cement and aluminum powder, natural fibers are added [33].

The composites containing diatomite described in the literature were produced using various techniques and intended for various applications, e.g., for medical purposes [32], agriculture [32], building materials [7,9], for removing heavy metal contaminants [8], or for ensuring the thermal efficiency of the phase change materials PCM [17]. Taking into account the potential use of materials containing diatomite, their various properties were tested. However, there are few literature reports regarding the study of sound absorption properties. It has been shown that in the case of bi-layered diatomite-based foams with a gradient structure and a diatomite content of 21 wt.%, the density of the material is very important. Low-density foams behave like porous materials, i.e., they show a sharp increase in sound absorption at medium frequencies and a high level of sound absorption at high frequencies. High-density foams show a low level of sound absorption in the entire tested range [16].

Diatomite/polyurethane composites were also fabricated, and the effects of diatomite, foam stabilizer, and plasticizer content on the microstructure, compressive strength, and sound absorption properties of diatomite/polyurethane composites were studied. The results show that the porous diatomite/polyurethane composites have good compressive strength and very good sound absorption properties. A diatomite/polyurethane composite containing 65 wt.% diatomite showed excellent sound absorption properties. The sound absorption peak was around 1600 Hz, and the sound absorption coefficient was greater than 0.9. The frequency range whose sound absorption coefficient was greater than 0.56 was more than 2000 Hz [34].

In scientific publications, one can also find research results related to composites based on epoxy resin with the addition of diatomite. One study showed that the composite containing 50 wt.% exhibits an increased elastic modulus of 2.9 MPa, satisfactory sound absorption properties at low frequencies with a modified mean sound absorption (MSAA) of 0.08 (for a sample only 5 mm thick) [35].

Although there are research results confirming the positive effect of diatomite on the acoustic properties of composites, little is known about the effect of diatomite processing and its degree of fragmentation on the sound absorption coefficient. This paper presents a comprehensive and innovative study of the effect of different fractions of diatomite and the effect of its thermal treatment on the sound absorption coefficient of flax/PLA/diatomite composites. To date, no research of this type has been conducted, and how to optimize acoustic parameters by controlling the degree of fragmentation and calcination of diatomites has not been determined. The presented research results make an important contribution to the development of this field of science and may contribute to the wider use of diatomites as materials to support sound absorption. The presented research results are also innovative due to the nature of this material, as the diatomites found in Poland are very different from other such materials, and their effects on sound absorption capabilities are also different. Thanks to the research, it was possible to determine for the first time whether the calcination process of diatomite affects sound absorption capacity, and it was determined whether the degree of fragmentation also has a significant effect. These are very important issues not only from an economic point of view (grinding and calcination costs) but also because of the environmental impact (energy consumption). The results of the presented research are important not only for scientific development but also for the possibility of commercialization in solutions such as panels and sound barriers, vehicle cabin enclosures, etc. The sound absorption capacity of materials used for various types of enclosures, shielding, etc., is important because of the need to protect the health of people working, for example, in the cabins of motor vehicles such as excavators, cranes, drilling rigs, etc. The development of such materials can contribute to the development of solutions to prevent excessive noise in the working environment. In addition, such materials should also be good thermal insulators, which is why this article presents the results of thermal conductivity tests for different variants of diatomite processing.

The conducted research adds to the existing knowledge on the use of diatomites as a filler in composites used for acoustic insulation. So far, the effect of thermal treatment of diatomite on the ability to dampen sound has not been described in the scientific literature, nor has the study of diatomite from the deposit presented in the article, which is quite significant due to the amount of material and its origin.

## 2. Materials and Methods

The study examined the possibilities of using diatomite grains as a factor in increasing the sound absorption of the material. For this purpose, several variants of the composite material have been developed, differing in terms of type, size, and amount of grain. Two types of diatomite were used, i.e., “non-calcined (raw)-gray” and “calcined-red”, three fractions of each type of diatomite differing in grain diameter, i.e., fine (0 ÷ 0.063 mm), medium (0.5 ÷ 3 mm), and coarse (2 ÷ 5 mm). The weight share of diatomite in the composite material was 25 wt.% or 50 wt.%. The percentage of diatomite by weight was selected based on the literature [16,35]. The assumption was to demonstrate the effect of the type of diatomite on the sound absorption of the composite with its share at the level of 25 wt.% and to determine whether and to what extent it will increase at the limiting share of diatomite at the level of 50 wt.%.

**Figure 3:**
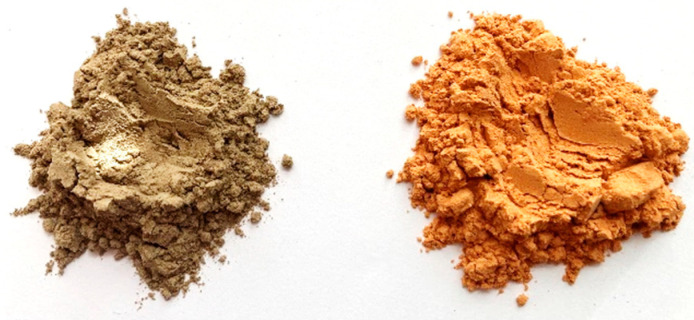
Diatomite used in the study (fraction 0 ÷ 0.063 mm) (raw, before thermal treatment—left side; calcined diatomite—right side).

The diatomite came from the only producer in Poland (Specialized Mining Company GÓRTECH Sp. z o.o., Cracow, Poland [36]), the diatomite mine in Jawornik Ruski (Podkarpackie Voivodeship). Calcination of the diatomite was carried out at 750 °C using a static method in a laboratory furnace for 24 h. The selection of the calcination temperature was based on the literature [4,37,38,39] and the authors’ previous studies. The temperature was chosen so that it was high enough for the processes of dehydration and dehydroxylation to occur, as well as the removal of organic substances, but not to exceed the temperature of mineral decomposition, and so that liquid phases (sintering) did not occur. Calcined diatomite at 750 °C exhibits optimal properties for damping vibrations due to its unique structural and compositional characteristics that arise from the calcination process. The thermal treatment of diatomite at this temperature enhances its mechanical strength and porosity, which are critical factors in vibration-damping applications. The thermal treatment at 750 °C significantly alters the microstructure of diatomite, leading to an increase in its specific surface area and porosity. This transformation is crucial as a higher porosity allows for better energy dissipation through the material when subjected to vibrational forces. The optimal pore structure formed at this temperature ensures that the material can absorb and dissipate vibrational energy efficiently, making it suitable for applications in construction and materials engineering. The thermal treatment enhances the material’s thermal stability and reduces its density, which further contributes to its ability to dampen vibrations effectively. The balance of porosity and strength achieved at this temperature is critical for applications where energy absorption and dissipation are necessary [40,41,42]. Figure 3 below shows the appearance of the diatomite used in the study.

The bulk density of the used diatomite in the loose and compacted state was performed according to PN-EN 1097-3:2000 [43]. The tests showed that the raw diatomite in the loose state has a bulk density of 0.82 g/cm^3^ (0 ÷ 0.063 mm); 0.85 g/cm^3^ (0.5 ÷ 3 mm); 0.68 g/cm^3^ (2 ÷ 5 mm), while after the calcination process at 750 °C, its bulk density in the loose state is, respectively, 0.78 g/cm^3^; 0.75 g/cm^3^; 0.65 g/cm^3^. The bulk density in the compacted state is 0.99 g/cm^3^; 0.97 g/cm^3^; 0.77 g/cm^3^ for the non-calcined material and 0.86 g/cm^3^; 0.81 g/cm^3^; 0.73 g/cm^3^ for the calcined material, respectively, for the individual fractions.

Diatomite from the company “GÓRTECH” meets the requirements of radiation hygiene and has a certificate issued by the National Institute of Hygiene (No. HR/B/34/2005). It can be used in buildings with rooms for human habitation or in buildings with livestock enclosures.

Oxide chemical composition analysis was performed for raw diatomite (before thermal treatment) and calcined at 750 °C, as shown in Figure 3. The oxide XRF analysis was performed on a SCHIMADZU EDX-7200 (SHIMADZU Europa GmbH, Duisburg, Germany). The test was carried out in an air atmosphere with holders designed for bulk materials and Mylar film, and the results are shown in Table 1. Only the most important oxides detected in the chemical composition, whose weight share is higher than 0.1, are shown in the table. The oxide composition of the two materials is similar.

**Figure 4:**
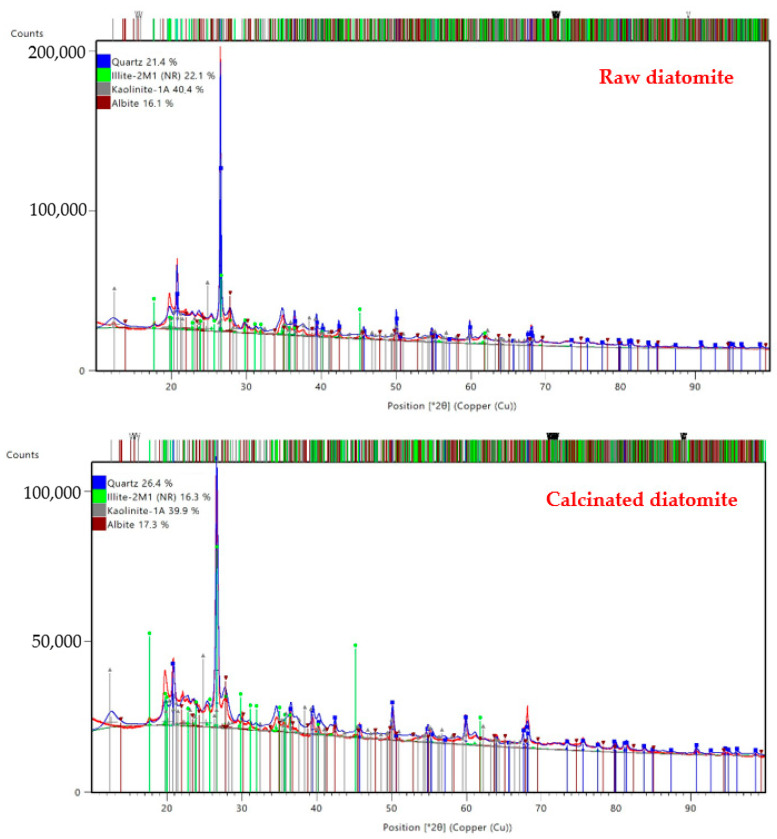
X-ray diffraction patterns of raw diatomite and calcined diatomite.

The mineralogical composition of diatomite was investigated by means of an X-ray diffraction technique using a PANalytical Aeris (Malvern Panalytical, Almelo, The Netherlands) diffractometer. X-ray diffractometry (XRD) is a technique that uses X-ray diffraction to determine the crystalline phases that make up the object under study. Quantitative analysis was performed using the Rietveld method, which was implemented in the HighScore Plus software (version 4.8). Diffractograms were recorded using Cu-Kα radiation in the scan range of 10–100° with a step of 0.003° (2θ) and a time per step of 340 s, using Cu Kα radiation. The X-ray diffraction patterns of raw diatomite and calcined diatomite are presented in Figure 4. Moreover, the results of the quantitative analysis are listed in Table 2. During the study, the weight content of mineral phases, including crystalline silica in the form of quartz, was determined.

In order to compare the sound absorption of different composite materials, they were manufactured in a way that ensured similar thickness of the test samples and the same external surface exposed to the sound wave. Components enabling biodegradation were used to produce the composite material containing diatomite. Polylactide (PLA) thermoplastic fibers 6.7 dtex/64 mm, under the name of Ingeo Fiber SLN2660D, with a finishing composition containing polylactide resin and no hazardous compounds, supplied by the Far Eastern Textile Ltd., Taiwan [44], were used as the composite matrix material. The melting point of PLA fibers is in the temperature range of 165 ÷ 170 °C. Waste standard flax fibers (flax) from Safilin Sp. z o.o., Poland [45], were used as a reinforcement for the composite.

Both types of fibers were combined in the appropriate proportion (PLA/flax—50 wt.%/50 wt.%) and mixed thoroughly, and then processed into a fleece, from which, after the needling process, a needle-punched nonwoven was created.

Samples of the composite material were produced on a hydraulic press with a water-cooling system from Hydromega Sp. z o. o., Poland. A layer system consisting of layers of nonwoven and layers of diatomite scattered between them was subjected to thermal pressing. In order to ensure a uniform surface of the composite material samples, the upper layer of the system was always made of nonwoven. A multilayer structure was closed in the form of a press, and the heating was turned on. The pressing temperature was 170 °C; the consolidation was carried out under a pressure of 0.58 MPa for 3 min. After the consolidation stage, the composite was cooled to room temperature using the water-cooling system. Each composite was manufactured from the system consisting of 75 wt.% of nonwoven (PLA/flax—50 wt.%/50 wt.%) and 25 wt.% of diatomite or 50 wt.% of nonwoven (PLA/flax—50 wt.%/50 wt.%) and 50 wt.% of diatomite. For a full comparative analysis of the sound absorption of materials containing diatomite, a reference material was also produced, i.e., without the addition of diatomite.

**Figure 5:**
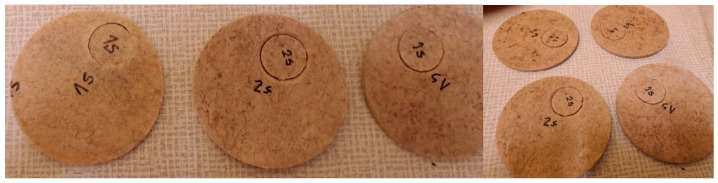
Examples of composites with the addition of diatomite.

Test samples for testing the sound absorption of composite material, with a thickness of approx. 5 mm were cut with steel dies, i.e., 29 mm diameter circles for high frequencies and 100 mm diameter circles for high frequencies. The thickness of the specimens (5 mm) was chosen based on previous research achievements by other authors [35]. This parameter was selected in order to make the research results comparable to the works of other scientists. Figure 5 shows an example of composites with the addition of diatomite.

## 3. Experimental

### 3.1. Morphology Investigations

The morphology of the diatomite was observed using a scanning electron microscope JEOL IT200 (JEOL, Tokyo, Japan). The morphology evaluation focused mainly on identifying the porous structure of diatom carapaces.

### 3.2. Laser Particle Size Analysis

The particle size characteristics were performed via the particle size analyzer Anton-Paar PSA 1190LD (Anton-Paar, Graz, Austria). Five test measurements were taken for each material, and then the mean and standard deviation were calculated using Kalliope Professional software (version 2.22.1). The tests were conducted only for the 0 ÷ 0.063 mm fraction to obtain an idea of what the size distribution of the finest particles looks like.

### 3.3. Thermal Conductivity of Diatomite

Measurements of the thermal conductivity of diatomite were carried out on an HFM 446 plate apparatus (Netzsch, Selb, Germany). The instrument operates according to specialized standards such as ASTM C1784 [46], ASTM C518 [47], ISO 8301 [48], EN 12664 [49], and others. The conductivity range is 0.007 to 2.0 W/m × K, the measurement accuracy is ± 1–2%. Temperature regulation and control are verified by a Peltier system. The thermal properties of diatomite were determined using the described device based on the hot and cold plate method. Thermal conductivity was tested in the temperature range of 0–20 °C.

### 3.4. The Acoustic Properties of the Composite

**Figure 6:**
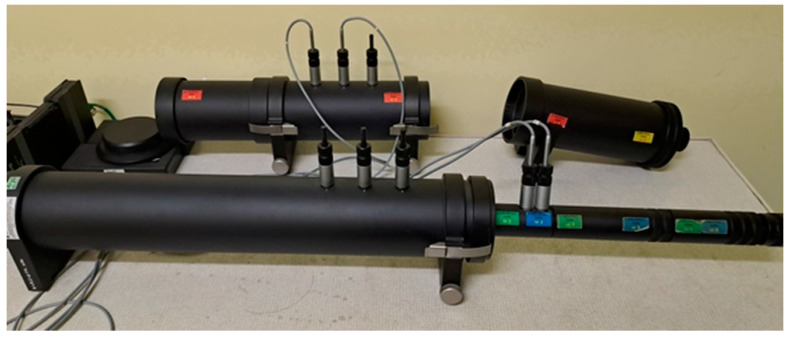
Impedance tube (Brüel & Kjaer, Virum, Denmark).

The acoustic properties of the composite materials were tested using an impedance tube, the so-called Kundt tube, type 4206, in the low-frequency range of normal incidence wave, from 50 Hz to 1600 Hz, and in the high-frequency range from 500 Hz to 6400 Hz. The sound absorption coefficient was determined in accordance with the PN-EN ISO 10534-2 standard [50] based on the following formula. Figure 6 shows the device used for testing to illustrate the test procedure.

$$\alpha =\frac{I_{a}}{I_{i}}$$

where:

α—sound absorption coefficient,
$I_{a}$—intensity of the sound absorbed,
$I_{i}$—intensity of the incident sound.

## 4. Results

### 4.1. Morphology Investigations

**Figure 7:**
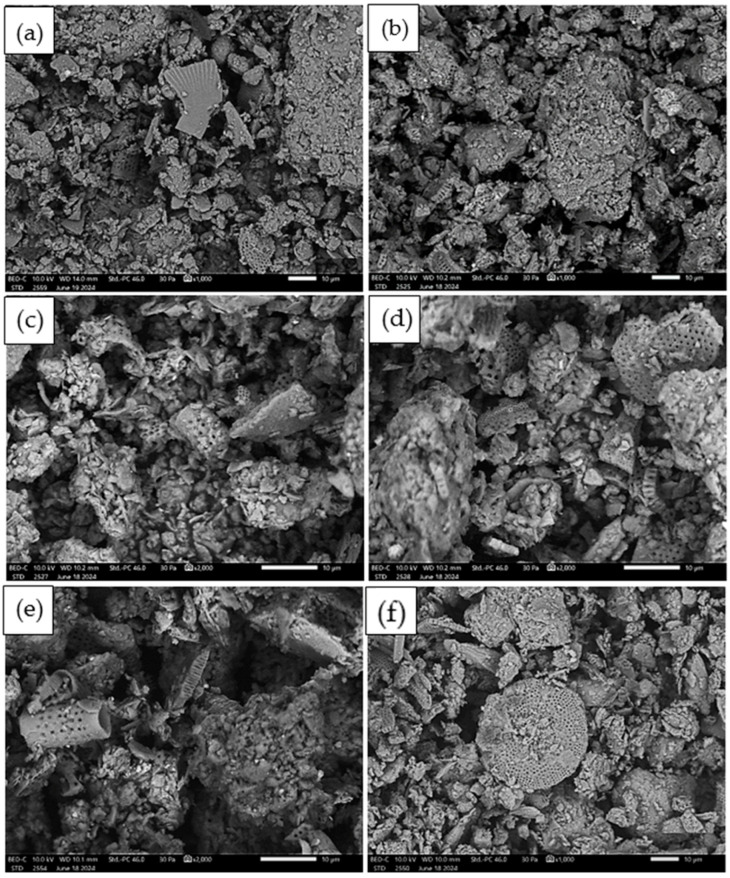
Morphology of diatomite particles: (**a**) 0 ÷ 0.063 mm without thermal treatment; (**b**) 0 ÷ 0.063 mm after thermal treatment; (**c**) 0.5 ÷ 3 mm without thermal treatment; (**d**) 0.5 ÷ 3 mm after thermal treatment; (**e**) 2 ÷ 5 mm without thermal treatment; (**f**) 2 ÷ 5 mm after thermal treatment.

Figure 7 below shows the morphology of diatomite particles for different fraction sizes without thermal treatment and after thermal treatment. The pictures show different forms of diatomite armor. Various shapes and sizes can be seen, as well as the porosity of these structures. The diatomite armor and its porous structure, which can be seen in the photos, are particularly important in terms of sound attenuation ability. The microstructure of diatomite may play a key role not only in terms of binding diatomite particles to the matrix but also because of its sound absorption ability. SEM images show forms of diatomite—disk-shaped, cylindrical (tubular), and mechanically degraded (crushed) form. It can be seen that after thermal treatment, the appearance of the diatomite particles did not change. Based on SEM observations, it is difficult to say whether there have been significant changes in the morphology of the diatomite particles after calcination. The photos were taken at a magnification of 1000 and 2000×. The marker in the figures corresponds to 10 μm.

### 4.2. Laser Particle Size Analysis

Table 3 shows the particle size distribution for the diatomite used in the study. Results for raw material and calcined material are presented. It was found that the calcination process caused a slight increase in the average particle diameter. This may be due to the agglomeration of the finest particles by partial sintering of the low-melting phases present in the diatomite. During the particle size distribution study, ultrasound was used to eliminate the risk of agglomeration of diatomite in aqueous media. The increase in the size of the measured particles after calcination was most likely due to permanent bonding due to thermal treatment and the occurrence of a liquid phase.

### 4.3. Thermal Conductivity of Diatomite

Table 4 shows the results of heat conduction coefficient tests for different fractions of diatomite before and after the calcination process. The results show that the calcination process reduces the thermal conductivity coefficient. The highest values of the heat conduction coefficient were recorded for diatomite with a fraction of 0.5–3 mm and the lowest for 0–0.063 mm. The fraction with the largest particle size had an intermediate thermal coefficient value. For the use of diatomite in sound-absorbing composites that can be used, for example, as covers or fillings in the cabins of heavy construction equipment (vehicles such as excavators, cranes, etc.), thermal insulation is just as important as sound absorption capacity (due to the operation of such equipment in different environmental conditions and at different temperatures).

### 4.4. The Acoustic Properties of the Composites

The sound absorption results for the flax/PLA/diatomite composites are presented in the figures below, showing the dependence of the sound absorption coefficient on the sound frequency.

#### 4.4.1. Tests in the Frequency Range of 50–1600 Hz

**Figure 8:**
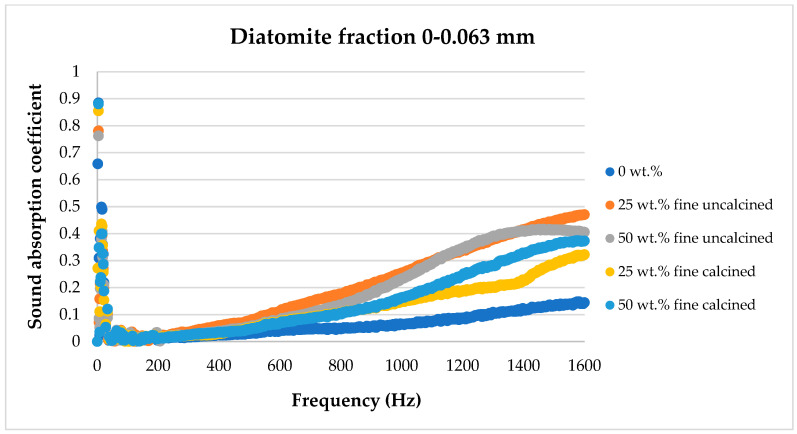
Effect of diatomite addition on the sound absorption coefficient of composites in the low-frequency range—diatomite fraction 0–0.063 mm.

**Figure 9:**
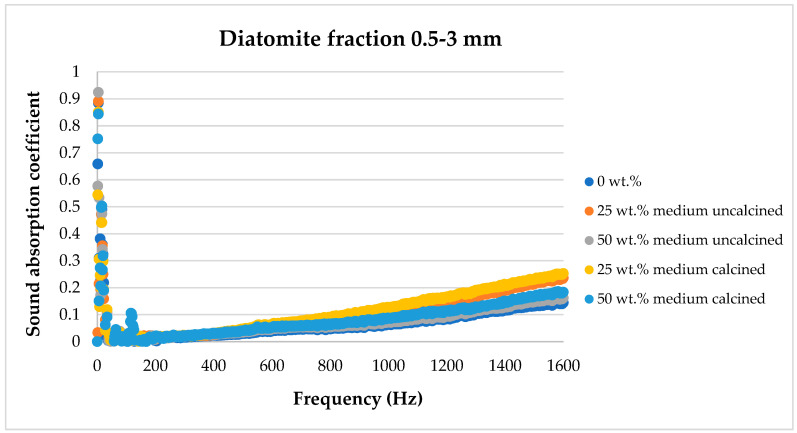
Effect of diatomite addition on the sound absorption coefficient of composites in the low-frequency range—diatomite fraction 0.5–3 mm.

**Figure 10:**
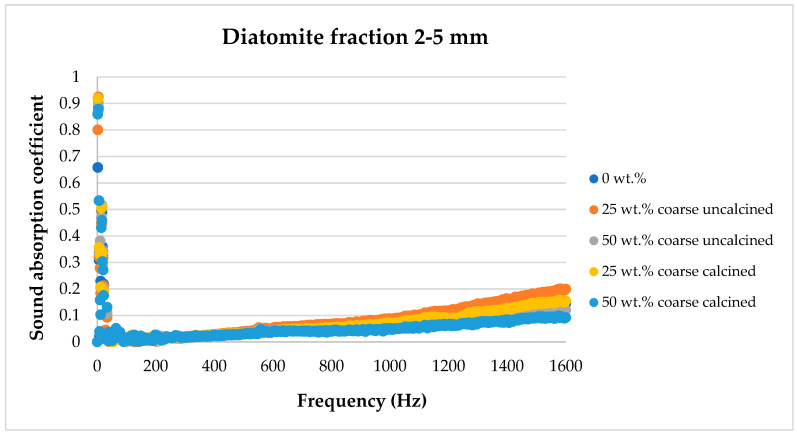
Effect of diatomite addition on the sound absorption coefficient of composites in the low-frequency range—diatomite fraction 2–5 mm.

**Figure 11:**
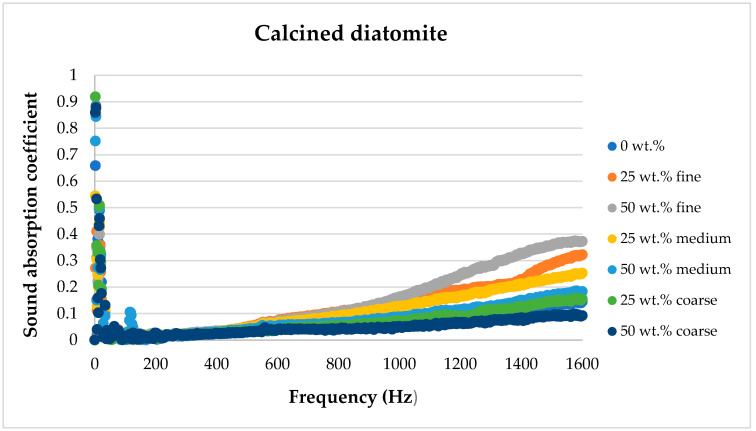
Comparison of the effect of diatomite addition on the sound absorption coefficient of composites in the low-frequency range for calcined diatomite.

**Figure 12:**
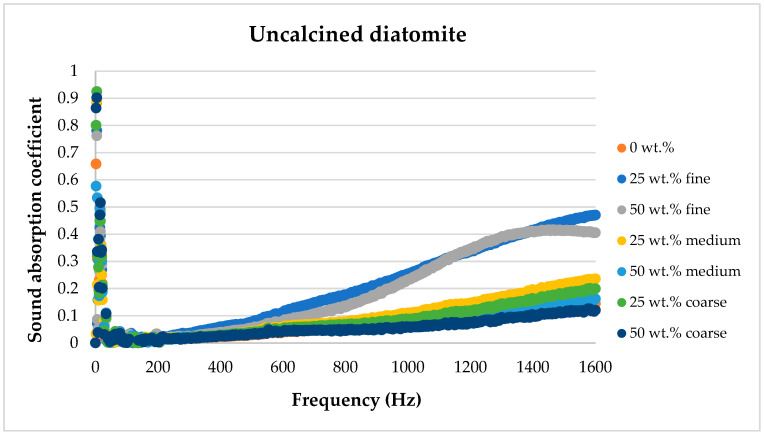
Comparison of the effect of diatomite addition on the sound absorption coefficient of composites in the low-frequency range for uncalcined diatomite.

Figure 8, Figure 9, Figure 10, Figure 11 and Figure 12 below show a comparison of sound absorption coefficients for composites with different additions of calcined and non-calcined diatomite. The purpose of the study was to determine how the calcination process of diatomite affects the sound absorption capacity of composites with it, as well as how the degree of fineness of the diatomite affects this coefficient.

As can be seen from the tests presented above in Figure 9 for diatomite with a fraction of 0–0.063 mm, the best characteristics in terms of sound absorption coefficient were obtained for the material containing 25 wt.% in non-calcined form. The composite containing 50 wt.% of the same diatomite had similar properties. Samples with calcined diatomite showed a lower sound absorption coefficient. Comparing the graph for the sample unmodified with diatomite, it can be seen that the addition of 25 wt.% of non-calcined diatomite with a particle size of less than 63 μm results in a more than three-fold improvement in acoustic properties.

The tests shown in Figure 10 illustrate the effect of diatomite with particle sizes in the range of 0.5–3 mm on the sound absorption coefficient of the composites. In this case, the most favorable properties were obtained for composites containing 25 wt.% diatomite (both calcined and non-calcined). It is noticeable, however, that compared to the graph presented above (Figure 9) and composites containing diatomite of smaller size, the addition of much larger diatomite particles already has a much less favorable effect than diatomite in dust form.

The addition of diatomite with a particle size of 2–5 mm has little effect on the sound absorption coefficient of the composites. As in the case of smaller fractions, the 25 wt.% diatomite addition also has the most favorable effect.

Comparing different fractions of diatomite and their different shares, for calcined diatomite, it can be concluded that in the frequency range of 50–1600 Hz, the 25 wt.% and 50 wt.% of diatomite addition in the form of 0–0.063 mm fine dust is the most favorable. The use of diatomite with larger fractions affects the sound absorption capacity to a lesser extent and may even negatively affect this parameter. The use of a 50 wt.% diatomite additive with a fraction of 2–5 mm causes a deterioration of the composite’s sound attenuation ability compared to the material without the introduction of diatomite.

The use of a non-calcined diatomite additive yielded the best results when the finest fraction below 63 μm was used. The addition of both 50 wt.% and 25 wt.% resulted in a several-fold improvement/increase in sound absorption coefficient.

#### 4.4.2. Tests in the Frequency Range of 500–6400 Hz

**Figure 13:**
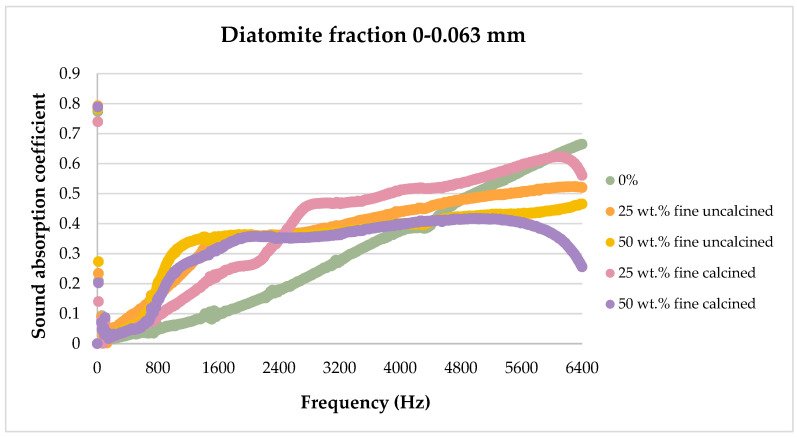
Effect of diatomite addition on the sound absorption coefficient of composites in the high-frequency range—diatomite fraction 0–0.063 mm.

**Figure 14:**
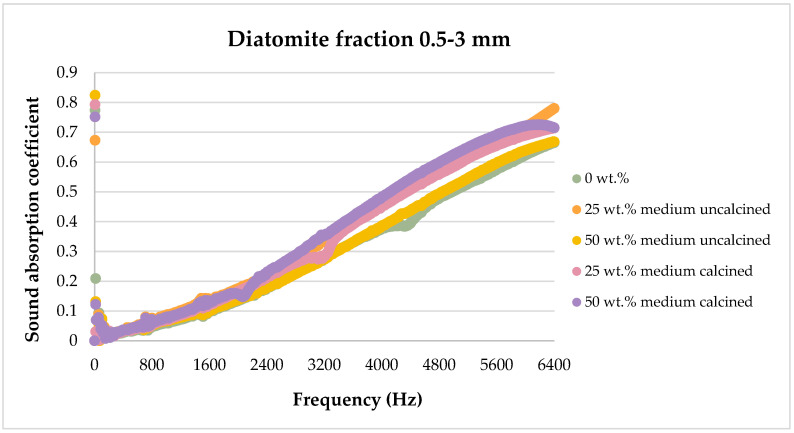
Effect of diatomite addition on the sound absorption coefficient of composites in the high-frequency range—diatomite fraction 0.5–3 mm.

**Figure 15:**
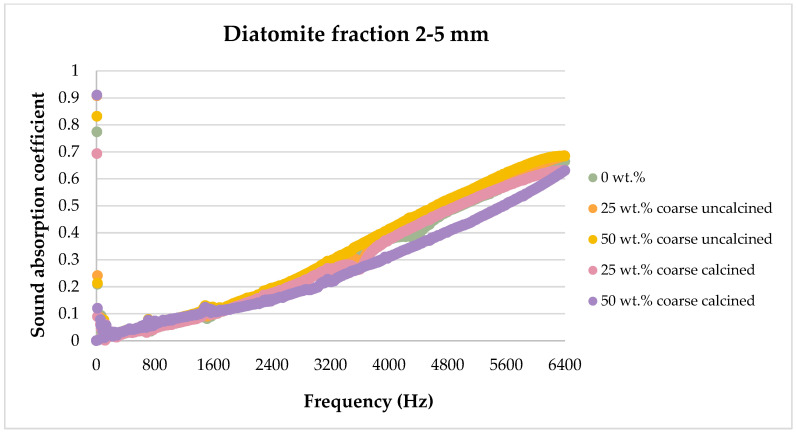
Effect of diatomite addition on the sound absorption coefficient of composites in the high-frequency range—diatomite fraction 2–5 mm.

**Figure 16:**
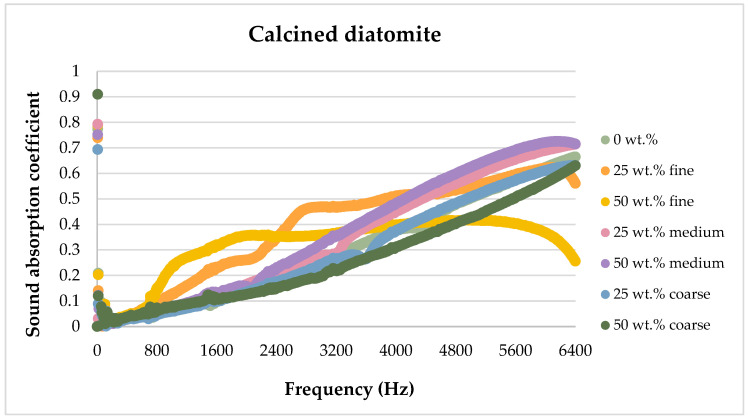
Comparison of the effect of diatomite addition on the sound absorption coefficient of composites in the high-frequency range for calcined diatomite.

**Figure 17:**
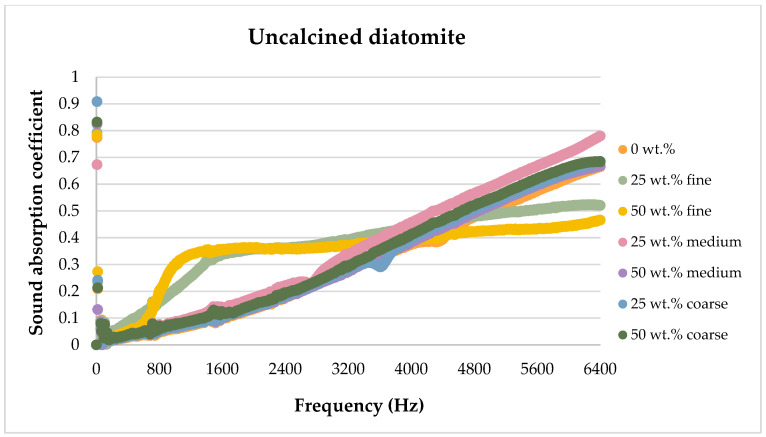
Comparison of the effect of diatomite addition on the sound absorption coefficient of composites in the high-frequency range for uncalcined diatomite.

Figure 13, Figure 14, Figure 15, Figure 16 and Figure 17 below present the results of sound absorption coefficient tests in the frequency range of 500–6400 Hz for composites with diatomite in both calcined and non-calcined forms. The three fractions of diatomite were used as before.

In the frequency range studied, the addition of the smallest size diatomite did not significantly improve sound absorption capacity. However, it was observed that the addition of both calcined and non-calcined diatomite in the amount of 25 wt.% and 50 wt.% affected the sound absorption capacity in the frequency range from about 800 Hz to about 4500 Hz. Beyond the 5000 Hz frequency range, the addition of diatomite had a negative effect. No significant differences were observed in terms of the effect of heat treatment or the mass proportion of the additive.

The use of larger diatomite fractions also did not yield positive results in the frequency range tested.

Analyzing the effect of diatomite on sound attenuation in the frequency range of 500–6400 Hz, one can see its positive effect in the range of 800–3500 Hz for both the calcined and untreated variants.

## 5. Discussion

Diatomites and diatomaceous earth are widely used as filter materials, sorbents, carriers for plant protection products and catalysts, thermal insulation, and polishing materials. Recently, the use of diatomite in construction binders has been increasingly investigated [37,38,51], and technologies are being developed to improve their sorption properties in terms of their ability to absorb petroleum substances [4,39]. Diatomites and diatomaceous earth have been used as ceramic fillers in paints and varnishes for many years. The use of diatomite as a filler in anti-corrosion coatings, as well as in other types of coatings or plastics, can bring a number of benefits. According to research carried out by certified masters of radiesthesia, diatomite of Polish origin is a material/additive for neutralizing the negative effects of geopathic (earth) radiation in the B-S-Pc range of the radiesthesia spectrum. This diatomite has a selective effect as it does not suppress the natural bands of radiation in the Cz-UF range that are positive for humans and animals.

It is also known for its antibacterial and fungicidal properties. Research by the authors of this article has shown that diatomite has growth-inhibiting properties against the bacteria tested: Bacillus subtilis, Escherichia coli, and two strains of Staphylococcus aureus. Diatomite showed a linear mycelial growth-limiting effect against the fungus species tested, Aspergillus niger. The results of this research will be presented in a separate scientific article.

The results presented in the paper showed that diatomite from Polish deposits is characterized by SiO_2_ content not exceeding 80 wt.%. The results of these studies are consistent with the work of other scientists [5,38]. Analysis of mineral phases revealed the presence of crystalline phases such as quartz, illite, kaolinite, and albite in the diatomite, which also coincides with the findings of other researchers [39,52,53]. Studies of diatomite morphology have shown that diatomite has a very porous structure, a highly desirable effect in terms of acoustic capabilities [54,55,56]. A particle size analysis of diatomite powder showed that the calcination process caused an increase in the particle size of the material. This increase was most likely due to permanent binding due to thermal treatment and the presence of a liquid phase [4,38,51]. The thermal treatment of diatomite also caused a decrease in the thermal conductivity coefficient for each of the fractions tested. The smallest diatomite particles obtained the lowest thermal conductivity coefficient after calcination [57]. The calcination process has a positive effect on lowering the thermal conductivity coefficient.

The acoustic properties of diatomite composites are significantly influenced by both thermal conductivity and porosity. Diatomite, a naturally occurring siliceous sedimentary rock, is characterized by its high porosity and low thermal conductivity, which are critical factors in determining its acoustic behavior. The unique microstructure of diatomite, composed of numerous interconnected pores, contributes to its sound absorption capabilities. As porosity increases, the ability of the material to attenuate sound waves also improves, leading to enhanced acoustic performance [40,58]. The relationship between porosity and acoustic properties can be attributed to the mechanisms of sound wave propagation through porous materials. Increased porosity generally results in a greater surface area for sound wave interaction, which can lead to higher sound absorption coefficients. For instance, studies have shown that diatomite composites with porosities exceeding 90% exhibit notable sound insulation properties, as the air trapped within the pores acts as a damping medium for sound waves [59]. Furthermore, the acoustic performance is also affected by the size and distribution of the pores; larger and more uniformly distributed pores tend to enhance sound absorption [35]. On the other hand, thermal conductivity plays a crucial role in the thermal management of diatomite composites, which can indirectly influence their acoustic properties. The thermal conductivity of diatomite is relatively low, which is beneficial for applications requiring thermal insulation. However, when diatomite is incorporated into composites with materials that have higher thermal conductivity, such as expanded graphite or carbon nanotubes, the overall thermal conductivity of the composite can be significantly enhanced [60,61]. This modification can lead to changes in the microstructural interactions within the composite, potentially affecting its acoustic properties. For example, the incorporation of conductive fillers can alter the density and stiffness of the composite, which may influence how sound waves propagate through the material [62]. Additionally, the interplay between porosity and thermal conductivity can create a complex relationship affecting the acoustic performance of diatomite composites. High porosity typically correlates with lower thermal conductivity, which can enhance sound absorption. However, if the thermal conductivity is increased through the addition of conductive materials, it may lead to a denser structure that could diminish the sound absorption capabilities [35,59]. Therefore, optimizing the balance between porosity and thermal conductivity is essential for achieving desired acoustic properties in diatomite composites. In conclusion, the effects of thermal conductivity and porosity growth on the acoustic properties of diatomite composites are interrelated. Increased porosity generally enhances sound absorption, while thermal conductivity influences the microstructural characteristics of the composite. The careful selection and combination of materials can lead to improved acoustic performance, making diatomite composites suitable for various applications in sound insulation and thermal management.

A number of these properties make diatomites an extremely attractive material that can be used as a filler or active additive in various types of plastics. The results presented in this article show that properly prepared diatomite can contribute to an increase in the sound attenuation coefficient and can be used in a variety of applications.

The studies conducted and presented above indicate that the calcination process is not valid for the use of diatomite in PLA-based composites to achieve better sound attenuation performance. The calcination of diatomite is most relevant for its use as a sorbent for petroleum substances. For other applications, the calcination process is not as important and may not be economically or environmentally viable. The studies presented above have also shown that the most beneficial way to improve sound attenuation capacity is to use diatomite with the smallest possible fraction.

The results obtained for the tested fiber composites with the addition of diatomite confirmed literature reports on similar composites with the addition of cellulose sub-microfibers or cellulose ultra-short and ultra-fine fibers. As the literature indicates [63,64], the content of sub-microfibers or ultra-short and ultra-fine fibers with a diameter of approx. 11 μm, which are thinner than the standard fibers used as reinforcement material in the composite, results in an adequately increased sound absorption coefficient of the composites. This effect is greater if the standard fibers are replaced by thinner fibers, in which case there is a larger fiber surface area involved in the interaction with the sound wave. Using diatomite particles of the smallest possible size, i.e., smaller than the transverse dimension of the standard fibers (the length of the elementary flax fiber varies on average between 1 mm and 120 mm, and the diameter also varies and ranges from 9.6 μm to 201.6 μm), the effect is similar. In our study, the finest fraction of diatomite, i.e., 0–0.063 mm (up to 63 μm), raw, i.e., with a smaller average particle size than after calcination, proved to be the best fraction. As indicated by the results of the study (Table 3), the average particle size for raw diatomite is 13.315 μm, while for calcinated diatomite, it is 17.240 μm.

## 6. Conclusions

Several conclusions can be drawn from the above discussion of the research results to conclude the study work as follows:

- Diatomite from Polish deposits is characterized by SiO_2_ content not exceeding 80 wt.%. In diatomite, there are crystalline phases, such as quartz, illite, kaolinite, and albite;
- The thermal treatment caused an increase in the particle size of the material. This increase was most likely due to permanent binding due to thermal treatment and the presence of a liquid phase;
- The smallest diatomite particles obtained the lowest thermal conductivity coefficient after calcination. The calcination process lowers the thermal conductivity coefficient. When diatomite is used in sound-absorbing composites, thermal insulation is as important as sound absorption capacity (due to the operation of such equipment in different environmental conditions and at different temperatures);
- The smallest fraction of diatomite particles and variants without thermal treatment give the best effect in terms of sound absorption. The calcination process does not positively affect the improvement of acoustic properties.

The most important research finding in this study is that the calcination process does not positively affect acoustic properties. So far, the thermal treatment of diatomite has been a very desirable process, as it significantly improved the sorption capacity of the material. For acoustic insulation, calcination is not necessary. This effect is very surprising and has a very positive impact on both environmental and financial aspects. Thermal treatment involves additional costs of electricity consumption, which can be avoided when producing diatomite composites for acoustic applications.

Different fractions of diatomite from the only mine of this raw material in Poland were tested. Diatomite was used as a filler in PLA-based composites with the addition of fibers, and the effect of diatomite on the sound-damping capacity of such prepared composites was studied. Heat-treated calcined and untreated material was used, and the following proportions of diatomite were used: 0 wt.%, 25 wt.%, and 50 wt.%. As a result of the tests carried out, positive preliminary results were obtained on the sound-absorbing properties of the composites with the addition of diatomite. Some variants of the produced composites, as well as the results of the obtained tests, can be useful in the design of vibro-acoustic protection, such as sound-absorbing enclosures, building partitions, acoustic screens, and others.

## Figures and Tables

**Table 1:** Oxide analysis for diatomite.

Type of Diatomite	Oxide Composition (wt.%)						
	SiO_2_	Al_2_O_3_	Fe_2_O_3_	K_2_O	SO_3_	TiO_2_	CaO
Raw diatomite	78.985	15.896	2.623	1.375	0.403	0.350	0.280
Calcinated diatomite	79.594	15.415	2.463	1.402	0.402	0.351	0.280

**Table 2:** Quantitative analysis of raw diatomite and calcined diatomite.

Type of Diatomite	Quantitative Share of Phase [%]			
	Quartz	Illite	Kaolinite	Albite
	01-075-8321	00-026-0911	00-058-2001	00-001-0739
	SiO_2_	Al_2_Si_3_AlO_10_(OH)_2_	Al_2_Si_2_O_5_(OH)_4_	NaAlSi_3_O_8_
Raw diatomite	21.4	22.1	40.4	16.1
Calcined diatomite	26.4	16.3	39.9	17.3

**Table 3:** Particle size distribution of diatomite.

Material	D_10_ [μm]	D_50_ [μm]	D_90_ [μm]	Average Particle Size [μm]	Standard Deviation [μm]
Raw diatomite	2.536	10.633	25.991	13.315	0.398
Calcinated diatomite	3.948	14.488	31.578	17.240	0.075

**Table 4:** Thermal conductivity of diatomite.

Type of Diatomite	Thermal Conductivity Coefficient λ [W/m × K]
Raw diatomite 0–0.063 mm	0.07725
Calcinated diatomite 0–0.063 mm	0.06969
Raw diatomite 0.5–3 mm	0.15385
Calcinated diatomite 0.5–3 mm	0.12656
Raw diatomite 2–5 mm	0.13260
Calcinated diatomite 2–5 mm	0.11845

## Data Availability

The original contributions presented in the study are included in the article, further inquiries can be directed to the corresponding authors.
